# 

**Published:** 1885-09

**Authors:** 


### BOOKS RECEIVED.

*A Text-Book of Medical Physics*, by John C. Draper, M.D., LL.D. Philadelphia: Lea Brothers & Co. Chicago: Jansen, McClurg & Co.

*Cholera*, by Alfred Stillé, M.D., LL.D. Philadelphia: Lea Brothers & Co. Chicago: Jansen, McClurg & Co.

*A Text-Book of Physiology*, by M. Foster, M.A., M.D., F.R.S. Third American from the fourth revised English edition. Philadelphia: Lea Brothers & Co. Chicago: Jansen, McClurg & Co.

*A System of Practical Medicine*, by American authors. Edited by William Pepper, M.D., LL.D. Vol. II. General Diseases (continued) and Diseases of the Digestive System. Philadelphia: Lea Brothers & Co. Chicago: Jansen, McClurg & Co.

*Elements of Modern Medicine*, by R. French Stone, M.D. New York: D. Appleton & Co. Chicago: Jansen McClurg & Co.

*The Technology of Bacteria-Investigation*, by Charles L. Dolley, M.D. Boston: S. E Cassino. Chicago: Jansen, McClurg & Co.

*Inebriism, a Pathological and Psychological Study*, by T. L. Wright, M.D. Columbus, Ohio: W. G. Hubbard. Bellefontaine, Ohio: Dr. T. L. Wright.

*Diseases of the Tongue*, by Henry F. Butlin, F.R.C.S. Philadelphia: Lea Brothers & Co. Chicago: Jansen, McClurg & Co.

*A Treatise on Epidemic Cholera and Allied Diseases*, by A. B. Palmer, M.D., LL.D. Ann Arbor, Michigan: Register Publishing House.

*Renal and Urinary Affections*, by W. Howship Dickinson. New York: William Wood & Co. Chicago: W. T. Keener.

*Poisons: Their Effects and Detection*, by A. W. Blyth. Vols I and II. New York: William Wood & Co. Chicago: W. T. Keener.

*A Practical Treatise on Diseases of the Kidneys and Urinary Derangements*, by Charles Henry Ralfe, M.A., M.D. Philadelphia: Blakiston, Son & Co. Chicago: W. T. Keener.

*An Introduction to the Study of the Diseases of the Nervous System*, by Thomas Granger Stewart, M.D. Philadelphia: J. B. Lippincott & Co. Chicago: W. T. Keener.

*Cancer: A Study of Three Hundred and Ninety-Seven Cases of Cancer of the Female Breast*, by Willard Parker, M.D. New York: G. P. Putnam's Sons. Chicago: W. T. Keener.

*The Treatment of Opium-Addiction*, by J. B. Mattison, M.D. New York: G. P. Putnam's Sons. Chicago: W. T. Keener.

*Comparative Anatomy and Physiolog* , by F. Jeffrey Bill, A.M., M.D. Philadelphia: Lea Brothers & Co. Chicago: Jansen, McClurg & Co.

### PAMPHLETS RECEIVED.

Report of the Proceedings of the Tennessee State Board of Health.

Bacterial Pathology. By Watson Cheyne.

Deviation of the Nasal Septum. By J. W. Gleitsmann, M.D.

Surgical Hemorrhage. By J. W. Gleitsmann, M.D.

Third Annual Report of the Provincial Board of Health of Ontario.

A Case of Poisoning Resulting from Chloroform taken Internally. By Llewllyn Eliot, M.D.

Shadows in the Ethics of the International Medical Congress. By Levi Cooper Lane, A.M., M.D.

Voice in Singers. Carl H. Von Klein, A.M., M.D.

Proceedings and Addresses at a Sanitary Convention held at Lansing, Mich., March 19th and 20th, 1885. ———, Dec. 2 and 3, 1884. ———, Apr. 17 and 18, 1884.

Co-operation of Citizens in Preventing the Spread of Disease. By Rev. W. A. Masker.

The Public Health Service of Michigan. By Hon. John Avery, M.D.

Transactions of the Louisiana State Medical Society.

An Address on Cholera Infantum. By Wm. Berry Watson, A.M., M.D.

Duty of the State Towards the Medical Profession. By Conrad George, M.D.

2011

LIBRARY INDEXED S.G.O.

SUBSCRIPTION PRICE, $3.00 PER YEAR.

Whole Number, 309. SEPTEMBER, 1885. Vol. LI., Number 3

INDEX MEDICUS

### THE CHICAGO MEDICAL JOURNAL AND EXAMINER

EDITORS:

S. J. JONES, M.D., LL.D.

W. W. JAGGARD, A.M., M.D. HAROLD N MOYER, M.D.

COLLABORATORS.

CHICAGO:

Professor J. A. ALLEN, M. D., LL. D.
Professor E. ANDREWS, M. D., LL. D.
Professor W. H. BYFORD, A. M., M. D.
Professor O. C. DeWOLF, A. M., M. D.
Professor E. C. DUDLEY, A. B., M. D.
Professor J. H. ETHERIDGE, A. M., M. D.
Professor C. FENGER, M. D.
Professor J. N. HYDE, A. M., M. D.
Professor E. F. INGALS, A. M., M.D.
Professor H. A. JOHNSON, M. D., LL. D.
CHARLES GILMAN SMITH, A. M., M.D
J. F. TODD, M. D.

MILWAUKEE, WISCONSIN:
Professor N. SENN, M, D.

WASHINGTON, D. C:
S. O. RICHEY, M. D.

UNITED STATES NAVY
MEDICAL DIRECTOR J. M. BROWNE, M. D.

CHICAGO:

